# CRISPR/Cas System: A Potential Technology for the Prevention and Control of COVID-19 and Emerging Infectious Diseases

**DOI:** 10.3389/fcimb.2021.639108

**Published:** 2021-04-23

**Authors:** Ronghua Ding, Jinzhao Long, Mingzhu Yuan, Yuefei Jin, Haiyan Yang, Mengshi Chen, Shuaiyin Chen, Guangcai Duan

**Affiliations:** ^1^ College of Public Health, Zhengzhou University, Zhengzhou, China; ^2^ Hunan Provincial Key Laboratory of Clinical Epidemiology, Xiangya School of Public Health, Central South University, Changsha, China; ^3^ Key Laboratory of Molecular Medicine in Henan Province, Zhengzhou University, Zhengzhou, China

**Keywords:** CRISPR/Cas, SARS-CoV-2, pathogen detection, clinical therapy, drug and vaccine development

## Abstract

The continued global pandemic of coronavirus disease 2019 (COVID-19) poses a serious threat to global public health and social stability and it has become a serious global public health problem. Unfortunately, existing diagnostic and therapeutic approaches for the prevention and control of COVID-19 have many shortcomings. In recent years, the emerging CRISPR/Cas technology can complement the problems of traditional methods. Biological tools based on CRISPR/Cas systems have been widely used in biomedicine. In particular, they are advantageous in pathogen detection, clinical antiviral therapy, drug, and vaccine development. Therefore, CRISPR/Cas technology may have great potential for application in the prevention and control of COVID-19 and emerging infectious diseases in the future. This article summarizes the existing applications of CRISPR/Cas technology in infectious diseases with the aim of providing effective strategies for the prevention and control of COVID-19 and other emerging infectious diseases in the future.

## Introduction

Coronavirus disease 2019 (COVID-19) caused by severe acute respiratory syndrome coronavirus-2 (SARS-CoV-2) first broke out in Wuhan, China, and rapidly grew into a global pandemic, seriously endangering human health and social development. By February 23, 2021, SARS-CoV-2 had become a global pandemic in 180 countries and regions, with the cumulative number of infections exceeding 110 million and the cumulative number of deaths exceeding 2.48 million. More importantly, with the ongoing mutation of SARS-CoV-2 and the coming winter and spring, these numbers may continue to increase (Grubaugh et al., 2020; Korber et al., 2020). In response to the SARS-CoV-2 pandemic, it is urgent to strengthen pathogen detection capacity and accelerate the development of vaccines and specific antiviral drugs. At present, the traditional nucleic acid detection method, mainly quantitative real-time reverse transcription-polymerase chain reaction (qRT-PCR), is recommended by WHO and some medical departments to detect pathogens. However, some researchers found that the results are unstable, and the sensitivity is even as low as 42.10%, and the sample processing requirements are very strict (Bustin and Nolan, 2020; Tesija Kuna et al., 2021). More importantly, the therapeutic effect of antiviral drugs has not achieved the expected effect, and symptomatic treatment is still the main treatment at present (Khan et al., 2020). Vaccine development is still in the clinical evaluation stage (Folegatti et al., 2020; Gao Q. et al., 2020; Tu et al., 2020), it will take some time before it can be applied, and even the mutation of the virus may make the vaccine less effective than expected (Becerra-Flores and Cardozo, 2020; Li Q. et al., 2020). Moreover, the vaccine may not be able to meet the huge global demand.

CRISPR (clustered regularly interspaced short palindromic repeats) consists of a gene encoding a Cas-related protein and the CRISPR array, which is a repetitive sequence within the prokaryotic genome, an immune weapon produced by the struggle between bacteria and phages throughout the history of evolution (Bondy-Denomy et al., 2013; Koonin et al., 2017). Therefore, bacteria can protect themselves by using the CRISPR/Cas system to eliminate foreign invading phage genes. Since 1987, CRISPR/Cas was discovered by Ishino in *E. coli*, scientists have been developing several biotechnologies based on this basic principle (Ishino et al., 1987). The development and application of the CRISPR/Cas system have given new impetus to the development of life sciences and biotechnology (Knott and Doudna, 2018). Especially in medical science, CRISPR/Cas has made remarkable achievements in gene editing, nucleic acid detection, functional gene screening, and so on (Zhou et al., 2014; Kurata et al., 2018; Pickar-Oliver and Gersbach, 2019; Wang et al., 2019; Yan et al., 2019). Also, in recent years, scientists have used the CRISPR/Cas system broadly in the prevention and control of infectious diseases. In the article, we reviewed recent advances regarding the application of the CRISPR/Cas system in the prevention and control of COVID-19 and other infectious diseases, such as in molecular diagnostics, infectious disease treatment, the antiviral drug, and vaccine development. And provide effective strategies for the prevention and control of COVID-19 and other future emerging infectious diseases.

## Application of CRISPR/Cas in Pathogen Detection

### Inadequacy of Existing Pathogen Detection

Pathogen detection is crucial for the prevention and control of infectious diseases. Early identification of pathogens enables identification of the source of infection and timely quarantine, so that appropriate anti-infective protocols can be developed to improve patient prognosis, especially for severe emerging infectious diseases such as COVID-19. However, the current detection of SARS-CoV-2 is mainly by qRT-PCR, which is time-consuming and dependent on specialized equipment and operators, which may not be available in developing countries or remote rural hospitals, and is not suitable for field testing (Corman et al., 2020; Lu et al., 2020). Besides, limited sensitivity may prevent the detection of asymptomatic infected persons, or some patients during the time-window of viral replication, who are likely to contribute to further transmission without medical treatment (Li Y. et al., 2020; Younes et al., 2020; Yu et al., 2020). overcome the shortcomings of qRT-PCR technology, some experts have suggested using a combined antibody assay to detect SARS-CoV-2 (Li Z. T. et al., 2020). Although enzyme‐linked immunosorbent assay (ELISA) has been successfully used to detect SARS-CoV-2, specific antibodies need to be produced after more than 7 days of infection with the virus (Lin et al., 2020; Lisboa Bastos et al., 2020; Wang Q. et al., 2020). In addition, antibody detection can produce cross-reactivity, and people with poor immunity may produce fewer antibodies, which can affect test results (Liu W. et al., 2020). Furthermore, isothermal amplification detection technology has a great prospect of application because of its simple operation and can be applied to field detection. However, the current isothermal amplification technology has its inherent defects in sensitivity, specificity, and anti-interference. Therefore, highly sensitive and specific, field-applicable pathogen detection is necessary for the detection of emerging infectious diseases (**Table 1**).

**Table 1:** Available diagnostic methods for COVID-19.

Test	Detection type	Positive rate	Time	Characteristics	Limitations	References
qRT-PCR	nucleic acid detection	38%	>2h	Currently the most common detection method, simple quantitative detection.	Time-consuming, poor sensitivity	(Liu R. et al., 2020)
ELISA	Immunological detection	80.4%	>2h	Fast, easy to operate, low sample volumes	Cross-reactivity, Antibodies are produced about 7-10 days after infection.	(Liu W. et al., 2020)
RT-LAMP	nucleic acid detection	92.9%	40–60 min	Highly sensitive, fast, and convenient field testing	Prone to false positives	(Baek et al., 2020)
CRISPR-based detection	nucleic acid detection	96%	30-60min	Highly sensitive, high specificity, fast and convenient for field testing., low-cost	Not yet widespread and in clinical trials	(Patchsung et al., 2020)
ddPCR	nucleic acid detection	40%	NA	Absolute quantification, high sensitivity	Expensive equipment, poor accessibility, and complexity of the operation	(Suo et al., 2020)
CT Scan	Radiological Screening	NA	NA	Check for disease progression, easy access, rapid detection	Unable to identify the pathogen of infection, clinical assistant tests	(Dong et al., 2020)

### Application of CRISPR/Cas-Based Systems for Rapid, Accurate Point-of-Care Diagnostics

**Figure 1:**
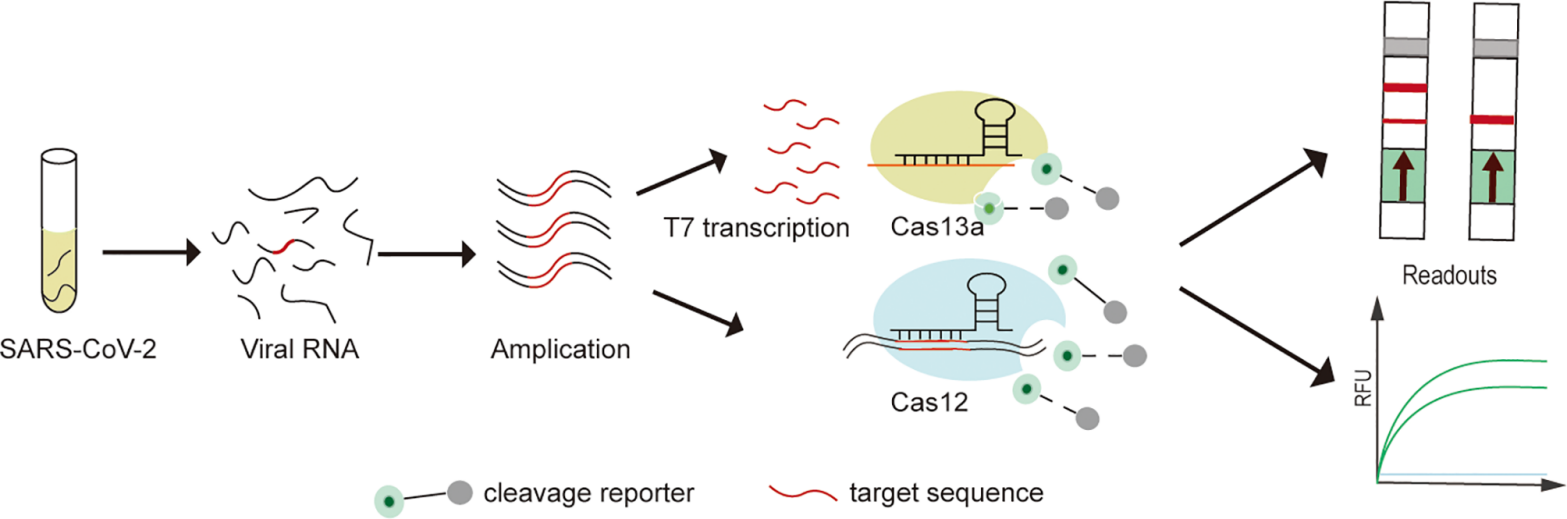
Experimental protocol for SARS-CoV-2 detection on CRISPR/Cas detection platform. (1) The extracted SARS-CoV-2 viral RNA is first pre-amplified (RT-RPA and RT-LAMP) into double-stranded DNA (dsDNA). (2) For Cas13a-SHERLOCK assay, dsDNA is first T7 transcribed into single-stranded RNA (ssRNA), followed by activation of the Cas13a cleavage reporter. (3) For Cas12a or Cas12b assays, direct detection of pre-amplified dsDNA enables activation of the Cas12a or Cas12b cleavage reporter. (4) Visualization of CRISPR/Cas assay results by fluorescence and lateral flow readouts.

In recent years, emerging nucleic acid detection technologies based on CRISPR/Cas developments have opened up new opportunities for pathogen detection. SHERLOCK (Cas13a), DETECTR (Cas12a), CDetection (Cas12b) and Cas14-DETECTR, which have successfully performed rapid, highly sensitive and accurate detection of a variety of pathogens (Gootenberg et al., 2017; Chen et al., 2018a; Chen et al., 2018b; Harrington et al., 2018; Teng et al., 2019) (**Figure 1**). Compared to traditional assays, CRISPR/Cas system has advantages in terms of rapidity, low cost, portability, ease of operation and extension, while maintaining high sensitivity and specificity (Zhou et al., 2018). In addition, the low sample quality requirements and high interference resistance mean that rapid sample pre-processing such as HUDSON can be combined to enable nucleic acid extraction on site without relying on specialized equipment (Gootenberg et al., 2017; Wang M. et al., 2020). In combination with lateral flow test strip technology for visualizing point-of-care diagnostics with the naked eye (Dai et al., 2020; Van Dongen et al., 2020). Fluorescence readout also can achieve point-of-care diagnostics with portable fluorescence collectors or colorimetric analysis (Yuan et al., 2020; Cheng et al., 2021).

CRISPR/Cas based detection of SARS-CoV-2 has been actively researched by scientists since the COVID-19 outbreak (**Table 2**). For example, James P. Broughton can successfully detect SARS-COV-2 within 30-40 minutes using the CRISPR-Cas12a-based method with a limit of detection (LoD) of up to 10 copies/µL, and the combination of lateral flow strip technology enables visible detection results in the field (Broughton et al., 2020b). Similarly, based on the CRISPR/Cas13a method, the SHERLOCK platform has been successfully validated in 534 clinical samples with a LoD of 42 copies/µL and 100% sensitivity for fluorescence readout and 97% for lateral-flow detection (Patchsung et al., 2020). Feng Zhang’s team developed the SARS-CoV-2 detection platform STOPCovid.v2 based on AapCas12b, which enables point-of-care diagnostics to be performed quickly and easily within 1 hour, without relying on specialized equipment. Also, it has obtained the FDA (US Food and Drug Administration) emergency use right of SARS-CoV-2 detection (Guglielmi, 2020; Joung et al., 2020). Besides, researchers using the Cas12a-based opvCRISPR platform were able to visualize fluorescence detection of SARS-CoV-2 with the naked eye in 45 min (Wang et al., 2021). It is worth noting that CRISPR/Cas technology allows not only rapid detection but also massively multiplexed nucleic acid detection. For example, Cheri M. Ackerman developed Combinatorial Arrayed Reactions for Multiplexed Evaluation of Nucleic acids (CARMEN) based on CRISPR/Cas13, which allows for the simultaneous differentiation of 169 viruses associated with humans, as well as the multiple identifications of various subtypes and even drug resistance mutations, thereby increasing detection rates while reducing detection costs (Ackerman et al., 2020).

**Table 2:** Summary of the CRISPR/Cas platform for SARS-CoV-2 detection.

Detection platforms	CRISPR/Cassystem	Limit of detection	Time	Characteristics	References
CREST	Cas13a	10copies/μL	30-50min	Combined with RT-PCR technology, CRISPR detection is stable. However, there may be drawbacks to field detection.	(Rauch et al., 2020)
SHERLOCK	Cas13a	42 copies per reaction	70min	Combination of RT-RPA amplification and lateral flow assay for rapid field testing.	(Patchsung et al., 2020)
SHINE	Cas13a	10copies/μL	50min	Improved sample pre-processing (HUDSON) combined with a one-step SHERLOCK for fast field inspection.	(Arizti-Sanz et al., 2020)
DETECTR	Cas12a	10copies/μL	45min	Combined with LAMP to solve the problem of unstable RPA amplification enables faster and more sensitive detection in the field.	(Broughton et al., 2020a)
STOPCovid	Cas12b	100 copy per reaction	<60min	Integrated LAMP and Cas12b detection enables one-step detection, making it easier and faster to operate.	(Joung et al., 2020)
CARMEN	Cas13a	attomolar	NA	Enables large-scale pathogen detection and simultaneous identification of 165 human-associated viruses.	(Ackerman et al., 2020)

Moreover, CRISPR/Cas technology has the unique ability to detect single nucleotide polymorphisms (SNP) compared to other above-mentioned detection technologies. Compared with conventional sequencing for SNP detection, CRISPR/Cas technology is simpler, faster, and cheaper, and more importantly, can detect low-frequency mutations. Faced with SARS-CoV-2 mutations such as the D614G and N501Y locus mutation. These mutations may increase the infectivity of SARS-COV-2 and even pose a significant threat to existing therapies and vaccines (Li Q. et al., 2020; Yurkovetskiy et al., 2020). So the identification of mutant strains based on high-specificity CRISPR/Cas technology has great significance. Although no researchers have yet performed CRISPR/Cas-based detection of the SARS-COV-2 mutation site, some scientists have successfully used Cas13a to detect the HBV drug resistance site rt204 (Gootenberg et al., 2017; Wang S. et al., 2020).

## CRISPR/Cas in Clinical Antiviral Therapy

### Application of Cas9 Gene-Editing to Clinical Antiviral Therapy

**Figure 2:**
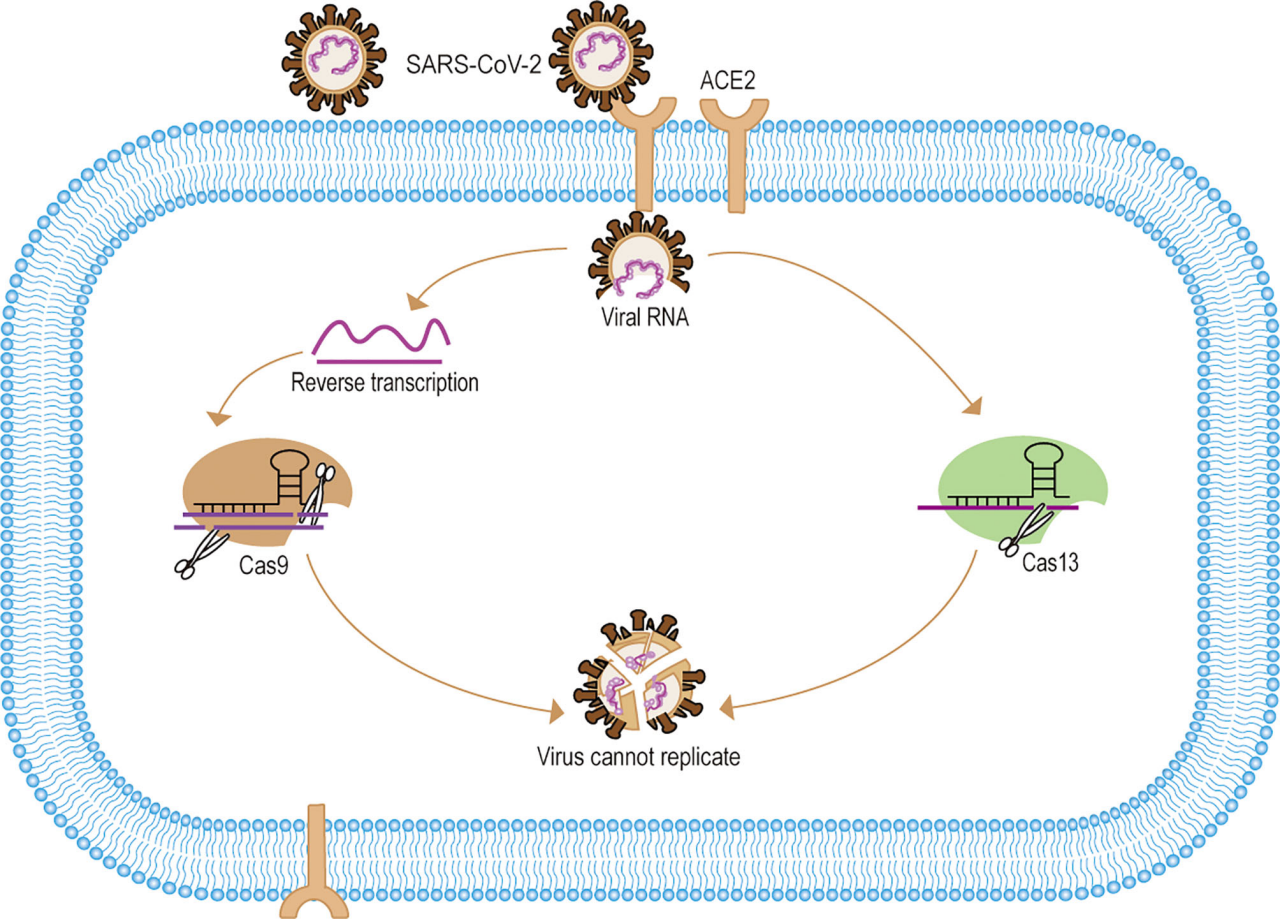
Mechanisms of CRISPR/Cas systematic elimination of SARS-CoV-2. (1) SARS-CoV-2 targets the ACE2 receptor to release viral RNA into cells. (2) After reverse transcription of the viral RNA to form dsDNA, the pre-designed sgRNA-Cas9 identified and specifically cleaved the viral DNA. (3) For Cas13 the system can directly identify and eliminate viral RNA. (4) The CRISPR/Cas system eliminates the SARS-CoV-2 gene, thus making the viral RNA unable to replicate and the viral gene unable to be expressed, and eventually making SARS-CoV-2 unable to replicate.

In the face of emerging infectious diseases such as SARS, MERS, and COVID-19, existing antiviral drugs are often ineffective. However, developing an effective antiviral drug is an arduous process. Therefore, clinical treatment is mainly supportive and to prevent complications, but in the absence of specific antivirals, this approach may result in a higher case fatality rate. The scientific community has been searching for ways to eliminate viruses more quickly and accurately, which could have significant implications for early source prevention and control of emerging infectious diseases. In recent years, experts using CRISPR/Cas9 gene-editing technology to treat many clinically refractory diseases. For one thing, the researchers used CRISPR/Cas9 targeting virus-specific sequences for gene editing to directly eliminate the virus to treat the disease. For example, The Edward M. Kennedy in the cell-free system trial used Cas9 to specifically target the HPV E6E7 gene to eliminate HPV infection to treat cervical cancer (Kennedy et al., 2014). Zhen has validated in animal studies that Cas9-targeted elimination of covalently closed circular DNA (cccDNA) is effective in the treatment of hepatitis B (Zhen et al., 2015). In addition, scientists also successfully used Cas9 to accurately eliminate Cytomegalovirus, Epstein-Barr virus infections *in vitro* (Ebina et al., 2013; Hu et al., 2014; Huo and Hu, 2019; Chen S. J. et al., 2020). For another thing, some researchers have used Cas9 to engineer T cells with the ability to treat specific diseases and thus indirectly eliminate viral infections (Zhao et al., 2018; Stadtmauer et al., 2020). For example, Cas9 was developed to improve CAR-T (Chimeric Antigen Receptor T-Cell Immunotherapy) therapy to treat incurable cancers such as B-cell leukemias and lymphomas (Brentjens et al., 2011; Porter et al., 2016; Eyquem et al., 2017; Labanieh et al., 2018). Moreover, many *in vivo* trial studies show Cas9 gene editing modifies CD4+ T cells to be effective in treating HIV infection, which may provide a new approach to refractory infectious diseases (Ebina et al., 2013; Liu et al., 2017; Hultquist et al., 2019; Xiao et al., 2019). With SARS-CoV-2 and other difficult to treat emerging infectious disease, CRISPR/Cas9 gene editing is likely to be one of the best treatments in the future (Grifoni et al., 2020) (**Figure 2**). In addition, with ongoing research and development, Cas12a and Cas14 also hold great potential for gene editing to treat infectious diseases in the future (Gao Z. L. et al., 2020).

### Cas13 Specifically Targets RNA Viruses for Degradation

Compared to Cas9, gene editing of Cas13-targeted RNA may offer a new approach to RNA virus therapy (Freije et al., 2019). LshCas13a system specifically targeted E6E7mRNA of HPV has a positive effect on inhibiting human cervical cancer *in vivo* testing (Chen Y. et al., 2020). Besides, Hao Li used Cas13a cleavage of the Dengue virus NS3 gene to efficiently inhibits viral replication *in vivo* testing (Li H. et al., 2020). Interestingly, Timothy R. Abbott used Cas13d to develop the PAC-MAN platform (prophylactic antiviral CRISPR in human cells) for viral inhibition that can effectively degrade RNA from SARS-CoV-2 sequences and live influenza A virus (IAV) in human lung epithelial cells. Notably, this approach provides a novel treatment strategy for SARS-CoV-2 and influenza virus, among others (Abbott et al., 2020; Nguyen et al., 2020) (**Figure 2**).

## Application of CRISPR/Cas Systems in Vaccine and Drug Development

### CRISPR/Cas9 Assisted Drug Development and Target Screening

**Figure 3:**
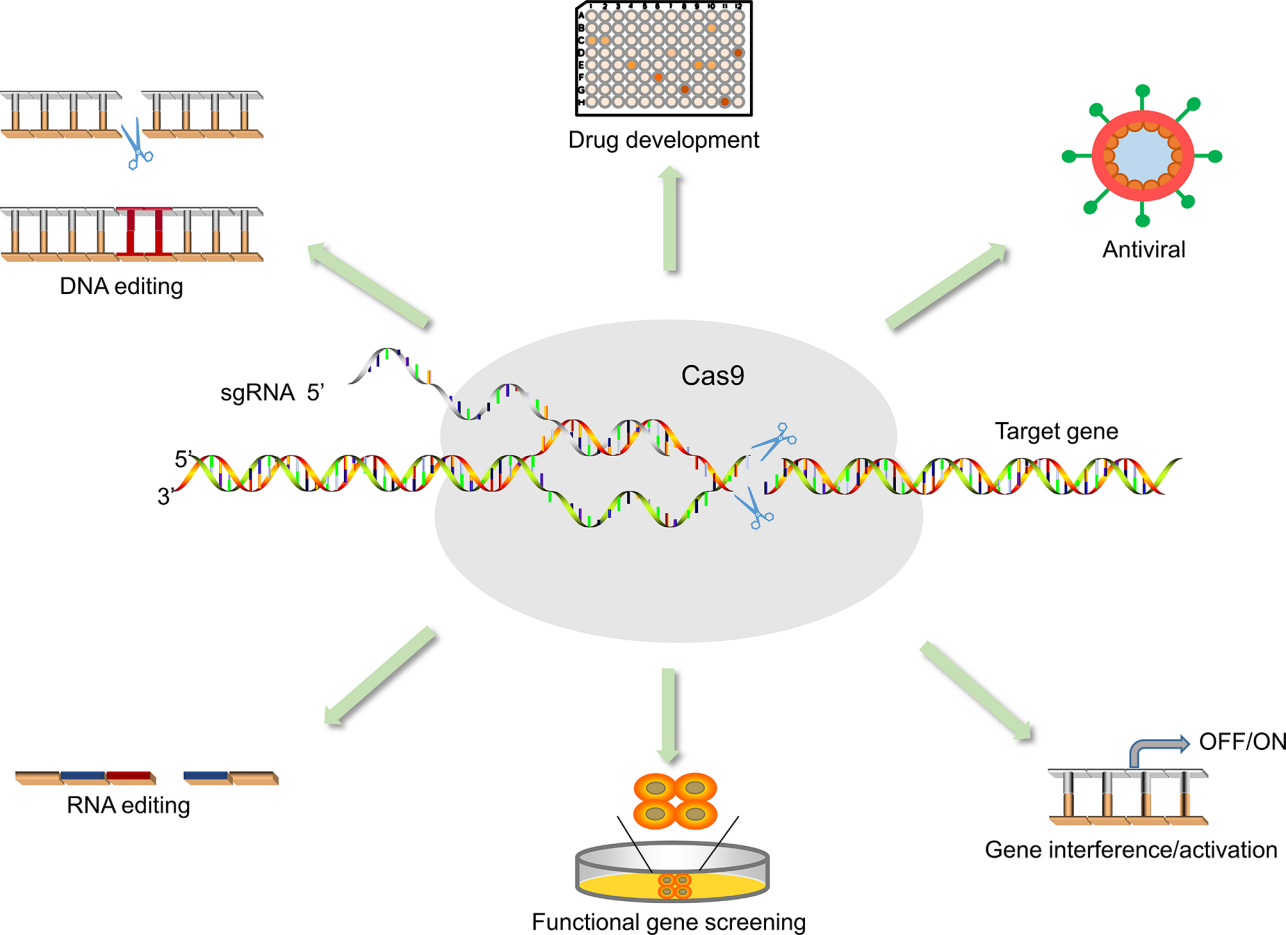
Application of CRISPR/Cas9 in medical sciences. The application of Cas9 is not only for antiviral but also for functional gene screening, drug development, animal model vector construction, and signaling pathway research.

The rapid development of effective antiviral drugs is also essential for the treatment of COVID-19 (**Table 3**). However, existing antiviral drugs are often ineffective in the treatment of emerging infectious diseases, mainly due to the lack of effective targets for the treatment of emerging viruses. Unfortunately, the main reason for the failure of antiviral drug development is also the difficulty in identifying effective targets, which makes development more difficult, expensive, and time-consuming. Moreover, host factors associated with viral action are critical to the success of treatment and drug development (Li B. et al., 2020; Wei et al., 2020). Therefore, how to identify functional targets more quickly and accurately is one of the challenges plaguing drug development. Although RNAi is undoubtedly a widely recognized and powerful method, it may produce misleading results due to off-target effects. As a result, the off-target effect may affect many functionally important genes (Settleman et al., 2018; Haley and Roudnicky, 2020). The emergence and development of CRISPR/Cas technology can more easily and quickly find potential drug targets to inhibit the replication of viruses, and provide safer and more feasible strategies for the treatment and prevention of diseases (Behan et al., 2019) (**Figure 3**). For instance, Flint used CRISPR/Cas technology to screen the genome-wide library and found that N-acetylglucosamine-1-phosphate transferase subunits alpha and beta (GNPTAB) were potential targets for the anti-Ebola virus (Flint et al., 2019). Interestingly, researchers on the bat cells of genome-wide screening explore the target of widespread antiviral treatment, after screening detects MTHFD1 targets is a potential target for broad-spectrum antiviral drugs. MTHFD1 inhibitor caprolactone broad antiviral activities against the Zika virus, mumps virus, and importantly, SARS-CoV-2 (Anderson et al., 2020). Furthermore, CRISPR/Cas screening can reveal host genes that regulate SARS-CoV-2 infection, such as SRSF protein kinases 1 and 2, ACE2, and HMGB1 (Heaton et al., 2020; Hoffmann et al., 2020; Wang R. et al., 2020; Wei et al., 2020). This provides insight into the signaling pathways underlying viral action. Although these articles have not been peer-reviewed, these ideas provide good insights and thoughts for exploring responses to emerging infectious diseases such as SARS-COV-2.

**Table 3:** Summary of current antiviral drugs or approaches to treat COVID-19.

Type	Candidate therapies	Mechanism of action	Clinical efficacy	References
Antiviral	Remdesivir	Inhibition of viral RNA replication	Efficacy is controversial, Under clinical trial for SARS-CoV-2	(Grein et al., 2020; Wang Y. et al., 2020)
Antiviral	Lopinavir-Ritonavir	Viral Protease Inhibitors, inhibits 3CLpro	Under clinical trial for SARS-CoV-2	(Cao et al., 2020)
Antiviral	Hydroxy-chloroquine, Chloroquine	Increasing endosomal pH prevents membrane fusion between virus and host cells	Under clinical trial for SARS-CoV-2	(Gautret et al., 2020; Pereira, 2020)
Antiviral	Favipiravir	RNA polymerase inhibitors	Under clinical trial for SARS-CoV-2	(Cai et al., 2020; Du and Chen, 2020)
Antiviral	PAC-MAN method based on CRISPR-Cas13d, CRISPR-Cas9	Specific degradation of SARS-COV-2	Although not in clinical trials, its unique advantages provide therapeutic strategies for refractory, poorly treated viruses	(Abbott et al., 2020)
Symptomatic therapeutic	Bevacizumab, SARS-CoV-2-Specific Neutralizing Antibodies	Attenuating the Inflammatory Response	Under clinical trial for SARS-CoV-2	(Thickett et al., 2001; Zhou and Zhao, 2020)

### Application of CRISPR/Cas9 in Vaccine Development

The prevention and control of outbreaks and epidemics of COVID-19 and other emerging infectious diseases require not only specific antiviral drugs but also vaccines. Vaccination is one of the most economical and effective public health interventions for the prevention and control of infectious diseases and one of the effective ways to prevent the large-scale spread of infectious diseases. However, the development of a vaccine is a long, complex process, and expensive. How to develop specific vaccines quickly and easily is a difficult problem for scientists. In response to emerging infectious diseases, CRISPR/Cas9 could boost progress in vaccine development. For example, Mustafa Ozan Atasoy developed and applied a highly efficient and rapid NHEJ-CRISPR/Cas9 (non-homologous end-joining, NHEJ) and Cre–Lox-mediated genome-editing approach for simultaneous deletion of virulence factors and insertion of antigens into the infectious laryngotracheitis virus to generate recombinant, multivalent, and safer vaccine vectors (Atasoy et al., 2019). Homology-directed repair (HDR) CRISPR/Cas9 and erythrocyte binding be used for the rapid generation of recombinant turkey herpesvirus-vectored avian influenza virus vaccines (Chang et al., 2019). In addition to Manuel used the CRISPR/Cas9 gene-editing system to produce recombinant African swine fever virus (ASFVs) (Borca et al., 2018). And the recombinant ASFV has great significance for the development of the live attenuated vaccine. Significantly, the researchers generated mouse models expressing human angiotensin-converting enzyme II (hACE2) using the Cas9 knock-in technique, which was used to study the transmission and pathogenesis of SARS-CoV-2 and to provide a useful tool for evaluating COVID-19 vaccines and therapeutic agents (Sun et al., 2020) (**Figure 3**).

## Limitations and Improvements of the CRISPR/Cas System

Despite the many advantages of the emerging CRISPR/Cas system mentioned above, it also has some limitations. For example, Cas9 may have limited specificity of the recognition site during gene editing, which may cause a high off-target effect and eventually lead to permanent damage to the genome, such as cancer (Fu et al., 2013). In addition, Cas12 and Cas9 also need to recognize protospacer adjacent motif (PAM) sequences before activation, and PAM sequences may limit targeting and affect editing efficiency and flexibility (Mojica et al., 2009; Marraffini and Sontheimer, 2010). More importantly, the transport vector of the CRISPR/Cas system is also a major problem (Komor et al., 2017; Glass et al., 2018). Currently, viruses are mainly used as transport vectors, which may cause potential harm to the human body. In addition, the human body may be immune to sgRNA and Cas proteins, thus affecting the proper role of the CRISPR/Cas system (Kim et al., 2018). Beyond that, it is noteworthy that the CRISPR/Cas system may have implications for medical ethics (Brokowski and Adli, 2019).

As the CRISPR/Cas system has been deeply researched, some limitations have been addressed. Jennifer Doudna’s team used the anti-CRISPR protein AcrllA4 to reduce the incidence of off-target effects by a factor of four, without any disruption to gene editing (Chen et al., 2017; Harrington et al., 2017; Jiang et al., 2019). In terms of optimizing the delivery carrier of the CRISPR/Cas system, scientists have made gradual progress in using nanocarriers to replace virus carriers (Lee et al., 2017). Another approach is to use smaller Cas proteins such as Cas14 (Harrington et al., 2018). Even more exciting is that although the CRISPR/Cas system needs to recognize specific PAM sequences, recent SCIENCE reports that using near-PAMless engineered can almost eliminate the limitations of PAM. Thus, high precision targeting can be realized in the application of genome editing, while further reducing off-target effect (Nishimasu et al., 2018).

## Conclusions

Although the clinical application of the CRISPR/Cas system is still in its infancy, its emergence provides many possibilities in the biomedical field. We should be more supportive and inclusive of emerging technologies. With continued in-depth research and clinical validation, we believe that the application of CRISPR/Cas system will play an irreplaceable role in the prevention and control of emerging infectious diseases in the future.

## Author Contributions 

RD and GD designed the study. RD and SC wrote the paper. JL, MY, YJ, HY, MC, and SC provided writing and revision suggestions. All authors contributed to the article and approved the submitted version.

## Funding

The work was supported by the National Science and Technology Specific Projects (2018ZX10301407), Hunan Provincial Key Laboratory of Clinical Epidemiology (2020ZNDXLCL003), and The Key Scientific Research Projects in Colleges and Universities of Henan Province (20A330004). Henan Province University Science and Technology Innovation Talent Projects (17HASTIT045).

## Conflict of Interest

The authors declare that the research was conducted in the absence of any commercial or financial relationships that could be construed as a potential conflict of interest.
